# Supplementary material for: The effects of genital myiasis on the diversity of the vaginal microbiota in female Bactrian camels
Source: BMC Vet Res. 2022 Mar 5;18:87. doi: 10.1186/s12917-022-03189-5 (PMC8897907; doi:10.1186/s12917-022-03189-5)
Supplement: Supplementary file 5 — Additional file 5. [file 12917_2022_3189_MOESM5_ESM.zip › MPL201709200_16s_yy/Treat1/css/js_css/jsPDF-master.zip!/jsPDF-master/docs/plugins_outline.js.html]

plugins/outline.js - Documentation


## Home

### Classes

- jsPDF

### Global

- addFont
- addHTML
- addMetadata
- addPage
- autoPrint
- CapJoinStyles
- circle
- ellipse
- getFontList
- lines
- lstext
- output
- rect
- roundedRect
- save
- setDisplayMode
- setDrawColor
- setFillColor
- setFont
- setFontSize
- setFontStyle
- setLineCap
- setLineJoin
- setLineWidth
- setPage
- setProperties
- setTextColor
- text
- triangle

# plugins/outline.js

```
/**
 * jsPDF Outline PlugIn
 * Copyright (c) 2014 Steven Spungin (TwelveTone LLC)  steven@twelvetone.tv
 *
 * Licensed under the MIT License.
 * http://opensource.org/licenses/mit-license
 */

/**
 * Generates a PDF Outline
 */
;
(function(jsPDFAPI) {
	'use strict';

	jsPDFAPI.events.push([
			'postPutResources', function() {
				var pdf = this;
				var rx = /^(\d+) 0 obj$/;

				// Write action goto objects for each page
				// this.outline.destsGoto = [];
				// for (var i = 0; i < totalPages; i++) {
				// var id = pdf.internal.newObject();
				// this.outline.destsGoto.push(id);
				// pdf.internal.write("<</D[" + (i * 2 + 3) + " 0 R /XYZ null
				// null null]/S/GoTo>> endobj");
				// }
				//
				// for (var i = 0; i < dests.length; i++) {
				// pdf.internal.write("(page_" + (i + 1) + ")" + dests[i] + " 0
				// R");
				// }
				//				
				if (this.outline.root.children.length > 0) {
					var lines = pdf.outline.render().split(/\r\n/);
					for (var i = 0; i < lines.length; i++) {
						var line = lines[i];
						var m = rx.exec(line);
						if (m != null) {
							var oid = m[1];
							pdf.internal.newObjectDeferredBegin(oid);
						}
						pdf.internal.write(line);
					}
				}

				// This code will write named destination for each page reference
				// (page_1, etc)
				if (this.outline.createNamedDestinations) {
					var totalPages = this.internal.pages.length;
					// WARNING: this assumes jsPDF starts on page 3 and pageIDs
					// follow 5, 7, 9, etc
					// Write destination objects for each page
					var dests = [];
					for (var i = 0; i < totalPages; i++) {
						var id = pdf.internal.newObject();
						dests.push(id);
						var info = pdf.internal.getPageInfo(i+1);
						pdf.internal.write("<< /D[" + info.objId + " 0 R /XYZ null null null]>> endobj");
					}

					// assign a name for each destination
					var names2Oid = pdf.internal.newObject();
					pdf.internal.write('<< /Names [ ');
					for (var i = 0; i < dests.length; i++) {
						pdf.internal.write("(page_" + (i + 1) + ")" + dests[i] + " 0 R");
					}
					pdf.internal.write(' ] >>', 'endobj');

					// var kids = pdf.internal.newObject();
					// pdf.internal.write('<< /Kids [ ' + names2Oid + ' 0 R');
					// pdf.internal.write(' ] >>', 'endobj');

					var namesOid = pdf.internal.newObject();
					pdf.internal.write('<< /Dests ' + names2Oid + " 0 R");
					pdf.internal.write('>>', 'endobj');
				}

			}
	]);

	jsPDFAPI.events.push([
			'putCatalog', function() {
				var pdf = this;
				if (pdf.outline.root.children.length > 0) {
					pdf.internal.write("/Outlines", this.outline.makeRef(this.outline.root));
					if (this.outline.createNamedDestinations) {
						pdf.internal.write("/Names " + namesOid + " 0 R");
					}
					// Open with Bookmarks showing
					// pdf.internal.write("/PageMode /UseOutlines");
				}
			}
	]);

	jsPDFAPI.events.push([
			'initialized', function() {
				var pdf = this;

				pdf.outline = {
					createNamedDestinations : false,
					root : {
						children : []
					}
				};

				var namesOid;
				var destsGoto = [];

				/**
				 * Options: pageNumber
				 */
				pdf.outline.add = function(parent,title,options) {
					var item = {
						title : title,
						options : options,
						children : []
					};
					if (parent == null) {
						parent = this.root;
					}
					parent.children.push(item);
					return item;
				}

				pdf.outline.render = function() {
					this.ctx = {};
					this.ctx.val = '';
					this.ctx.pdf = pdf;

					this.genIds_r(this.root);
					this.renderRoot(this.root);
					this.renderItems(this.root);

					return this.ctx.val;
				};

				pdf.outline.genIds_r = function(node) {
					node.id = pdf.internal.newObjectDeferred();
					for (var i = 0; i < node.children.length; i++) {
						this.genIds_r(node.children[i]);
					}
				};

				pdf.outline.renderRoot = function(node) {
					this.objStart(node);
					this.line('/Type /Outlines');
					if (node.children.length > 0) {
						this.line('/First ' + this.makeRef(node.children[0]));
						this.line('/Last ' + this.makeRef(node.children[node.children.length - 1]));
					}
					this.line('/Count ' + this.count_r({
						count : 0
					}, node));
					this.objEnd();
				};

				pdf.outline.renderItems = function(node) {
					for (var i = 0; i < node.children.length; i++) {
						var item = node.children[i];
						this.objStart(item);

						this.line('/Title ' + this.makeString(item.title));

						this.line('/Parent ' + this.makeRef(node));
						if (i > 0) {
							this.line('/Prev ' + this.makeRef(node.children[i - 1]));
						}
						if (i < node.children.length - 1) {
							this.line('/Next ' + this.makeRef(node.children[i + 1]));
						}
						if (item.children.length > 0) {
							this.line('/First ' + this.makeRef(item.children[0]));
							this.line('/Last ' + this.makeRef(item.children[item.children.length - 1]));
						}

						var count = this.count = this.count_r({
							count : 0
						}, item);
						if (count > 0) {
							this.line('/Count ' + count);
						}

						if (item.options) {
							if (item.options.pageNumber) {
								// Explicit Destination
								//WARNING this assumes page ids are 3,5,7, etc.
								var info = pdf.internal.getPageInfo(item.options.pageNumber)
								this.line('/Dest ' + '[' + info.objId + ' 0 R /XYZ 0 ' + this.ctx.pdf.internal.pageSize.height + ' 0]');
								// this line does not work on all clients (pageNumber instead of page ref)
								//this.line('/Dest ' + '[' + (item.options.pageNumber - 1) + ' /XYZ 0 ' + this.ctx.pdf.internal.pageSize.height + ' 0]');

								// Named Destination
								// this.line('/Dest (page_' + (item.options.pageNumber) + ')');

								// Action Destination
								// var id = pdf.internal.newObject();
								// pdf.internal.write('<</D[' + (item.options.pageNumber - 1) + ' /XYZ null null null]/S/GoTo>> endobj');
								// this.line('/A ' + id + ' 0 R' );
							}
						}
						this.objEnd();
					}
					for (var i = 0; i < node.children.length; i++) {
						var item = node.children[i];
						this.renderItems(item);
					}
				};

				pdf.outline.line = function(text) {
					this.ctx.val += text + '\r\n';
				};

				pdf.outline.makeRef = function(node) {
					return node.id + ' 0 R';
				};

				pdf.outline.makeString = function(val) {
					return '(' + pdf.internal.pdfEscape(val) + ')';
				};

				pdf.outline.objStart = function(node) {
					this.ctx.val += '\r\n' + node.id + ' 0 obj' + '\r\n<<\r\n';
				};

				pdf.outline.objEnd = function(node) {
					this.ctx.val += '>> \r\n' + 'endobj' + '\r\n';
				};

				pdf.outline.count_r = function(ctx,node) {
					for (var i = 0; i < node.children.length; i++) {
						ctx.count++;
						this.count_r(ctx, node.children[i]);
					}
					return ctx.count;
				};
			}
	]);

	return this;
})(jsPDF.API);
```

  

Documentation generated by JSDoc 3.4.2 on Sun Oct 09 2016 11:08:27 GMT+0100 (BST) using the docdash theme.
